# When graphite meets Li metal

**DOI:** 10.1093/nsr/nwaa120

**Published:** 2020-06-03

**Authors:** Yu Ding, Guihua Yu

**Affiliations:** Materials Science and Engineering Program and Department of Mechanical Engineering, The University of Texas at Austin, USA; Materials Science and Engineering Program and Department of Mechanical Engineering, The University of Texas at Austin, USA

Wetting, indicating the ability of a liquid to spread out over solid surfaces, is of vital importance in addressing the scientific issues related to energy and environment technologies. The study of wetting encompasses the academic disciplines of surface chemistry, nanotechnology, materials science and energy science. The past several decades have witnessed significant progress in achieving desirable wetting performance with water, which has resulted in broad technological applications, such as antifouling techniques, water–oil separation and water-harvesting [1]. In the field of energy storage, Li-ion batteries are widely used in portable electronics and electric vehicles, but the standard graphite anode is already near its theoretical capacity. Replacing graphite with Li metal (LM), the ‘holy grail’ anode with a high theoretical capacity of 3860 mAh/g, shows great promise in achieving more widespread applications [2]. However, LM suffers from low cycling efficiency, infinite volume change and uncontrollable dendrite growth. The method of using graphite to confine LM has been proved to be an effective solution. However, it is still challenging to composite LM with pure graphite directly, since carbon seems lithiophobic, and surface coating is largely required to improve the wettability of carbon with Li via ‘reactive wetting’ [3].

**Figure 1.**
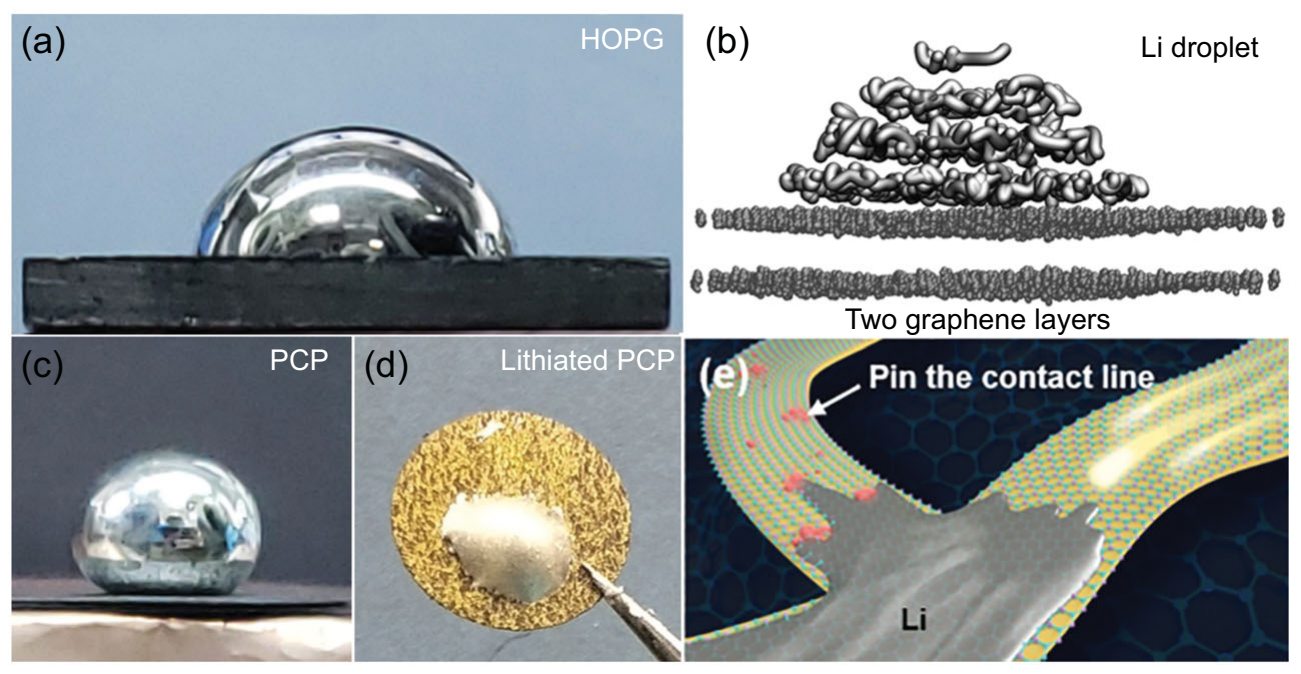
(a) Photograph of liquid Li droplet on HOPG with a small contact angle (CA). (b) *Ab initio* MD calculation of a Li droplet/graphene system at 500 K. (c) and (d) Digital photos of Li droplets on porous carbon paper (PCP) (c) and lithiated PCP (d). (a)–(d) are adapted from [4]. (e) Schematic of spreading Li metal on graphite and pining the contact line by surface contaminants.

Currently, it remains unknown whether graphite is essentially lithiophobic. To answer this fundamental question, recently Duan and co-workers carefully conducted contact angle (CA) measurements on several graphitic substrates and demonstrated that graphite is lithiophilic at low potentials free of contaminants [4]. They observed that highly ordered pyrolytic graphite (HOPG) immediately shows a CA of 73° with molten Li (Fig. 1a). The *ab initio* molecular dynamics simulation further proved that graphite is intrinsically lithiophilic (Fig. 1b). However, further experiments with porous carbon paper (PCP) showed that surface contaminants on graphite would pin the contact line, causing contact-line hysteresis and a large apparent CA (Fig. 1c). More interestingly, the oxidizing agents (air, moisture in the ambient environment) or the reducing agents (C_6_→LiC_6_ via a lithiation process) play a critical role in the wetting dynamics (Fig. 1c–e). In light of the unveiled wetting characteristics of various carbon materials, this study proposed a new approach for fabricating Li–PCP and Li–graphite powder composites with controllable Li/C ratio.

In closing, this work by the Luo, Li and Huang groups reveals for the first time the fundamental wetting mechanism between LM and graphite, and represents a significant step forward for compositing LM with carbon matrices in a controllable fashion. The new battery chemistry elucidated and the novel fabrication method proposed can be extended to other alkali metals, including Na and K, to promote the cell performance of the broad alkali ion batteries [5]. In the meantime, future research should focus on reducing the thickness of LM-based composite anodes and coupling them with traditional cathodes or Li-free cathodes and solid-state electrolytes towards higher energy density and better safety.

***Conflict of interest statement*.** None declared.
